# Development and Initial Validation of a Self-Scored COPD Population Screener Questionnaire (COPD-PS)

**DOI:** 10.1080/15412550801940721

**Published:** 2008-04-15

**Authors:** Fernando J. Martinez, Anastasia E. Raczek, Frederic D. Seifer, Craig S. Conoscenti, Tammy G. Curtice, Thomas D'Eletto, Claudia Cote, Clare Hawkins, Amy L. Phillips

**Affiliations:** ^1^Division of Pulmonary and Critical Care Medicine, University of Michigan Health System, Ann Arbor, MI, USA; ^2^QualityMetric Incorporated, Lincoln, RI, USA; ^3^Quillen College of Medicine, Johnson City, TN, USA; ^4^Clinical and Scientific Affairs, Boehringer Ingelheim Pharmaceuticals, Inc., Ridgefield, CO, USA; ^5^Health Economics and Outcomes Research, Boehringer Ingelheim Pharmaceuticals, Inc., Ridgefield, CO, USA; ^6^Pfizer Global Pharmaceuticals, New York, NY, USA; ^7^VA Medical Center, Bay Pines, FL, USA; ^8^San Jacinto Methodist Hospital, Baytown, TX, USA

**Keywords:** Pulmonary Disease, Chronic Obstructive, Spirometry, Health Survey, Questionnaire, Screening

## Abstract

COPD has a profound impact on daily life, yet remains underdiagnosed and undertreated. We set out to develop a brief, reliable, self-scored questionnaire to identify individuals likely to have COPD. COPD-PS™ development began with a list of concepts identified for inclusion using expert opinion from a clinician working group comprised of pulmonologists (n = 5) and primary care clinicians (n = 5). A national survey of 697 patients was conducted at 12 practitioner sites. Logistic regression identified items discriminating between patients with and without fixed airflow obstruction (AO, postbronchodilator FEV_1_/FVC < 70%). ROC analyses evaluated screening accuracy, compared scoring options, and assessed concurrent validity. Convergent and discriminant validity were assessed via COPD-PS and SF-12v2 score correlations. For known-groups validation, COPD-PS differences between clinical groups were tested. Test-retest reliability was evaluated in a 20% sample. Of 697 patients surveyed, 295 patients met expert review criteria for spirometry performance; 38% of these (n = 113) had results indicating AO. Five items positively predicted AO (*p* < 0.0001): breathlessness, productive cough, activity limitation, smoking history, and age. COPD-PS scores accurately classified AO status (area under ROC curve = 0.81) and reliable (*r* = 0.91). Patients with spirometry indicative of AO scored significantly higher (6.8, SD = 1.9; *p* < 0.0001) than patients without AO (4.0, SD = 2.3). Higher scores were associated with more severe AO, bronchodilator use, and overnight hospitalization for breathing problems. With the prevalence of COPD in the studied cohort, a score on the COPD-PS of greater than five was associated with a positive predictive value of 56.8% and negative predictive value of 86.4%. The COPD-PS accurately classified physician-reported COPD (AUC = 0.89). The COPD-PS is a brief, accurate questionnaire that can identify individuals likely to have COPD.

## INTRODUCTION

Chronic obstructive pulmonary disease (COPD) is a common disease with a profound impact on a patient's functioning and is underdiagnosed (1). A major objective of the Global Initiative for Chronic Obstructive Lung Disease (GOLD) is to increase awareness among healthcare providers and the general public of the significance of COPD symptoms (2). Despite the existence of evidence-based diagnostic and treatment guidelines, many patients with COPD continue to be undiagnosed or mis-diagnosed. COPD may remain undiagnosed for several reasons. Patients may not visit a physician because they have become accustomed to symptoms, may be concerned that their respiratory symptoms are self-inflicted, or are unsure whether COPD can be treated (3, 4). Additionally, spirometry is underutilized (5–7) and may be difficult to interpret by some healthcare providers (8, 9). Furthermore, asthma and COPD may be confused as they can both exhibit fixed airflow obstruction (AO) (10).

COPD screening that focuses only on patients meeting a limited set of characteristics, such as older smokers, may fail to detect COPD among individuals in the general population (e.g., younger adults with early disease) (11) or those having another etiology (e.g., occupational exposure). Similarly, identifying only individuals who are current smokers may miss those with COPD symptoms who have already quit smoking (11).

Hence, there is a need for a simple method to help identify persons who might have COPD. A simple, self-administered, self-scored tool to screen individuals for the disease may lead to increased awareness, earlier symptom recognition, and the use of spirometry for accurate diagnosis. Dissemination of such a tool in the general population might encourage individuals to discuss their respiratory symptoms with a healthcare provider. This tool could also assist physicians in identifying those individuals who would need spirometric assessment. Although several investigators have developed surveys for describing the impact of COPD, measuring the outcomes of treatment, or assessing disease severity, there are few tools designed to identify patients with COPD in a nonclinical setting (12).

We developed a simple, self-administered screening tool for the identification of patients with possible COPD in the general population. The COPD Population Screener™(COPD-PS™; QualityMetric Incorporated, Lincoln, Rhode Island, USA) is a 5-item questionnaire, designed to meet the following criteria: (1) a high correct classification rate for AO diagnosis while retaining a good trade-off between sensitivity and specificity; (2) self-scored, brief, and simple to complete; and (3) demonstrated adequate levels of reliability and preliminary evidence for validity. Of particular interest was the development of a tool appropriate for the general population rather than patients already seeking treatment for respiratory difficulties.

## MATERIALS AND METHODS

### Working Group and Survey Development

A clinician working group comprised of 5 pulmonary specialists, 4 primary care physicians, and 1 respiratory therapy professor was assembled to assist in the development of the COPD-PS questionnaire. The working group guided development of specific survey-question content, participated in designing a clinical development and validation study, and assisted in the evaluation of the study results.

Based on clinical experience, this working group first identified 7 conceptual domains relevant to the detection of COPD and easily identified by patients. A development survey included items that measured the conceptual domains in terms of presence, frequency, duration, or quality. An abbreviated description of these items is contained in Table 1, while a complete list of the items is enumerated in the Appendix. Additionally, a 12-item, short-form, general health-status assessment (SF-12®v2 Health Survey™ [QualityMetric Incorporated, Lincoln, Rhode Island, USA]) (13), demographic items, questions about resource use, and a chronic condition checklist also were employed.

**Table 1:** Item content of variables tested for inclusion in the COPD-PS™ questionnaire

Conceptual Domain	Abbreviated Item Content
Dyspnea	How much of the time short of breath?
	Out of breath with activity
	Short of breath at night
	Frequency of shortness of breath in last year
	Short of breath limits exercise
	Short of breath under specific conditions (e.g., lying down, getting dressed, climbing stairs, heavy exercise, etc.)
	Out of breath more than others of same age
Cough	Frequency of cough in last year
	Cough first thing in the morning
	Persistent cough that won't go away
	Cough that makes chest hurt
	Coughing “attacks” during exercise
	Cough that wakes at night
	Need to cough to clear chest
	Use of cough remedies
	Frequency of nagging cough
Phlegm	Frequency of coughing up “stuff,” such as mucus or phlegm
	Brought up phlegm or mucus first thing in the morning
	Need to clear chest of “stuff” in morning
Colds/bronchitis	Get a lot of chest colds
	Colds stay with me
	Colds last for weeks, not days
	Seem to catch a cold more easily than others Frequent bouts with bronchitis
	Get bronchitis at least once every winter
Chest Congestion/Wheezing	Feels like something stuck in chest or lungs
	Feeling of heaviness in chest
	Chest congestion
	Noisy breathing when sleeping
	Noisy breathing, gurgling, rattling during day
Functional Impact	Do less than used to because of breathing problems
	Breathing problems limit usual activities, enjoyment of life
	Difficulty performing work or other daily activities because of breathing problems
	Breathing problems limit usual activities
	Breathing problems kept from socializing
	Felt frustrated by breathing problems
	Breathing problems left too tired to do daily activities
	Breathing problems kept from getting as much done
	Breathing problems make it difficult to focus attention on other things
Personal Characteristics	Affected by strong smells, fumes
	Smoking history (current and past status, pack-years)
	Family history of lung disease
	Exposure to secondhand smoke at home, work
	Live with someone who smokes
	Exposed to dust, gases, etc., at work

COPD-PS: COPD Population Screener.

### Data Collection

To identify an optimal set of questions for inclusion in the COPD-PS questionnaire, a national cross-sectional survey of patients receiving care from 4 pulmonary specialists and 8 general practice sites was conducted during an 8-week period in 2004. Selected clinical sites were required to have experience performing spirometry following American Thoracic Society (ATS) standards (14, 15). Approval of the study protocol was obtained by each site using a central Institutional Review Board (Chesapeake Research Review, Inc.), and all patients provided written consent.

Patients aged 35 years and older with a previously scheduled office visit were recruited for study participation. Patients were excluded from the study for the following reasons: being allergic to bronchodilators, having a condition contraindicating study participation, participating in another clinical study, or currently seeking care for an acute respiratory problem. Based on a priori power calculations for logistic regression and sensitivity/specificity analyses (16–18), approximately 100 patients with COPD were sought.

Patients completed the development survey and physicians recorded patient demographics and medical history. Spirometry was performed before and 10 minutes after albuterol administration via metered-dose inhaler (90 μg, 2 puffs), according to ATS standards current at the time of the study (14) using predicted values from Morris et al. (19). For the purposes of this study, AO was defined based on GOLD guidelines (postbronchodilator forced expiratory volume in 1 second/forced vital capacity [FEV_1_/FVC] < 70%) (3) as airway obstruction that is not fully reversible. Patients with AO, identified by spirometry, were further characterized as having mild AO or moderate-to-very-severe AO.

Spirometry tracings were reviewed post hoc by an independent pulmonary specialist auditor to evaluate adherence to ATS standards for acceptability and reproducibility. Patients with spirometry tracings meeting the strict ATS criteria were included in the analysis. A random subsample of patients (20%) completed the development survey (but not spirometry) again 2 weeks later to generate test-retest reliability estimates; survey methods to optimize return rates were implemented (20).

### Statistical Analysis

Analyses were performed using SAS (Version 8.2 for Windows, SAS Institute Inc., Cary, NC, USA) and STATA (version 8.0; StataCorp LP, College Station, TX, USA).

### Item selection

Based on spirometry results, patients were categorized as “No AO” and “AO.” Point-biserial correlations of survey items with dichotomous AO diagnosis were generated. Forward stepwise logistic regression models were used to test blocks of items with similar content to identify the best predictors for AO status within each unidimensional set. Items within content blocks that met the criterion for significant model contribution (*p* < 0.10) were retained for inclusion in a second stage of modeling to identify the best multidimensional set of items (this significance level was selected to ensure items with any possible contribution to prediction were identified for further study). Items were evaluated as both continuous variables and as sets of dichotomized response options to study the contribution of the total item and specific response options.

Age and smoking variables (including pack-years, categorical smoking status, and other variables regarding smoking experience) were also tested to identify optimal questions. Based on overall model fit and significant contribution of individual items, a final multivariate logistic model was constructed. Consistency of these results was examined across specialist and general practice sites. The final model was applied to the total study sample (N = 697) to study consistency across different sample definitions, although the accuracy of the AO outcome in the full sample cannot be confirmed. To estimate the robustness of final model results, 1000 bootstrap samples were drawn with replacement from the original audited sample and analyzed.

### Scoring and screening accuracy

Three scoring options for combining item responses in the final model were evaluated: 1) total sum of item responses, 2) dichotomous variable for each item, and 3) weighted sum of item responses. Receiver operating characteristic (ROC) analyses (21) were conducted to evaluate the COPD-PS score in screening for AO. The area under an ROC curve (AUC), odds ratios, sensitivity and specificity, positive and negative predictive values, and percent correctly classified were estimated for the continuous screener score and at each scoring level or “cut-point.”

### Reliability

Test-retest reliability for the COPD-PS score was assessed with product-moment and intraclass (22, 23) correlations between scores at study entry and 2-week follow-up.

### Empirical validation

Clinician-working-group input assured a degree of content validity by defining conceptual domains and specific content items based on clinical experience. COPD is associated with decrements in quality of life (3); hence, convergent and discriminant validity was assessed by correlating COPD-PS scores and results from the SF-12v2 Health Survey. A series of known-groups construct validation analyses were conducted to examine the COPD-PS mean score differences between groups differing in the construct being measured (24). We hypothesized that patients with more respiratory problems would score significantly higher on the COPD-PS than those without for the categories of AO severity groups, site type, physician-reported COPD status, self-reported COPD status, work/school absenteeism due to breathing problems, self-reported use of bronchodilators, and overnight hospitalization for breathing problems. The ROC analyses for COPD-PS and CAO diagnosis were completed to examine concurrent validity of the COPD-PS screener.

## RESULTS

### Patient Characteristics

Of the 697 patients completing both the development survey and spirometry, 445 (64%) and 252 (36%) were from general practice and specialist sites, respectively. A total of 295 patients who had spirometry results meeting strict ATS standards were included in the analytic sample (105 [36%] from general practice; 190 [64%] from specialist sites). Table 2 presents patient characteristics by provider type. The average patient age for the total sample was 62.1 ± 13.0 years (range 35–91 years). Approximately 40% of the sample was male. Patients enrolled from specialist sites were older and more likely to be male, white, and less likely to be Hispanic.

**Table 2:** Patient characteristics by provider type

	Analytic Sample (N = 295) 100%	General Practice (N = 105) 36%	Pulmonologist (N = 190) 64%
Mean Age (SD)	62.1 (13.0)	58.2 (14.4)*	64.2 (11.6)*
Male (%)	40.0	32.4†	44.2†
Education (%)			
Less than high school	13.3	17.4	11.1
High school	24.1	15.3†	28.9†
Beyond high school	62.6	67.4	60.0
Employment (%)			
Full/part-time student	0.0	0.0	0.0
Working full/part-time	33.2	36.3	31.5
Retired/unemployed	49.3	42.2	53.3
Homemaker/other	17.5	21.6	15.2
Marital status: married (%)	63.0	54.8†	67.6†
Ethnic background (%)			
Black/African American	6.9	10.6	4.8
White	82.5	63.5*	93.1*
Asian/Pacific Islander	0.7	1.9	0.0
American Indian/Alaskan Native	0.7	1.0	0.5
Hispanic/Spanish/Latino	9.3	23.1*	1.6*
Smoking status (%)			
Current smoker	16.4	21.1	13.7
Never smoker	35.5	49.5*	27.9*
Former smoker	48.1	29.1*	58.4*
Chronic conditions (self-report, %)			
Arthritis	48.8	48.6	49.0
Hypertension	46.9	53.3	43.4
Rhinitis or sinusitis	39.7	43.3	37.8
Asthma	41.0	34.6	44.4
COPD	38.2	20.2*	48.2*
Other lung problems	28.3	8.8*	39.2*
Clinical depression	17.7	21.0	15.9
Diabetes	19.7	22.1	18.4
Cancer, except skin cancer	11.5	10.5	12.1
Congestive heart failure	11.9	12.4	11.6
Chronic conditions (clinician-report, %)			
Arthritis	43.1	45.7	41.6
Hypertension	42.7	51.4†	37.9†
Rhinitis or sinusitis	42.2	46.7	40.0
Asthma	36.4	32.7	38.5
COPD	39.1	21.0*	49.2*
Other lung problems	25.5	7.7*	35.3*
Clinical depression	17.4	21.9	14.8
Diabetes	16.7	21.9	13.8
Cancer, except skin cancer	9.5	10.5	9.0
Congestive heart failure	10.5	10.5	10.6
Effects of breathing problems (%)			
Missed 1+ days work/school in past 4 weeks	10.5	5.7	13.6
Hospitalized overnight in past 3 months	7.0	2.9†	9.3†
AO diagnosis: FEV_1_/FVC < 70% (%)	38.4	17.1*	50.3*

SD: standard deviation; COPD: chronic obstructive pulmonary disease; FEV_1_: forced expiratory volume in 1 second; FVC: forced vital capacity.

* *p* < 0.01

† *p* < 0.05.

Approximately 38% of patients had AO based on spirometry results, severity being mostly moderate-to-very severe (85%). Prevalence of AO differed by site type—17% and 50% of patients enrolled from general practice sites and specialist sites had AO, respectively (*p* < 0.0001). The mean pre- and postbronchodilator FEV_1_% predicted for patients with AO and without AO is shown in Table 3. In the AO group, the postbronchodilator mean FEV_1_% predicted was only slightly higher than the prebronchodilator mean FEV_1_% predicted, suggesting these patients had poorly reversible airway obstruction.

**Table 3:** Pre-and postbronchodilator administration mean (SD) FEV_1_%

	N (%)	Reversibility	Pre-BD Mean FEV_1_% predicted (SD)	Post-BD Mean FEV_1_% predicted (SD)
No AO:				
Pre-BD FEV_1_/FVC ≥ 70%	169 (58%)	—	91.1 (18.4)	93.0 (18.7)
Post-BD FEV_1_/FVC ≥ 70%				
Pre-BD FEV_1_/FVC < 70%	12 (4%)	41.7%	58.8 (31.3)	67.4 (32.1)
Post-BD FEV_1_/FVC ≥ 70%				
AO:				
Pre-BD FEV_1_/FVC < 70%	113 (38%)	10.6%	56.2 (24.2)	58.8 (26.1)
Post-BD FEV_1_/FVC < 70%				

Reversibility: ≥ 200 mL improvement and ≥ 12% improvement of baseline FEV_1_. SD: standard deviation; FEV_1_: forced expiratory volume in 1 second; BD: bronchodilator; COPD: chronic obstructive pulmonary disease; FVC: forced vital capacity.

### Questionnaire Item Selection

Responses to potential items for the COPD-PS questionnaire met basic criteria for Likert-type questions. Item responses were well distributed, and most items had a standard deviation (SD) of at least 1.0. Item-level missing data were less than 3% for all items, except shortness of breath with activity (8% missing). Correlations of the survey items with AO diagnosis ranged from 0.00 to 0.38. In the first stage of logistic models, 23 items were significant (*p* < 0.10) in predicting AO status.

A series of stepwise logistic regression analyses with the 23 items revealed that a combination of two symptom- and one functioning-based items best discriminated between patients with and without AO: shortness of breath frequency, production of sputum/phlegm frequency, and functional limitations due to breathing problems. Also the interaction of dyspnea and cough items was significantly predictive of AO. The same three variables performed the best in regression models with the total sample as well. Two items were significantly associated with AO,but in the negative direction. Awake at night with breathing problems and need to clear chest in the morning predicted a *lack* of AO but added little to the overall model fit. As such, only items with a positive association with AO were retained for the COPD-PS questionnaire.

Patient characteristics were added to the logistic model; age and smoking history improved AO prediction. Three versions of smoking variables were assessed: pack-years, categorical smoking status (i.e., current, former, never-smoker) and a single item regarding lifetime consumption of at least 100 cigarettes. The simple, single-item measure presented the best trade-off between explanatory power and ease of administration. Secondhand smoke exposure was evaluated regardless of individual smoking status and was nearly a significant predictor (*p* < 0.08), but did not improve overall model fit. The final logistic model with 5 variables produced significant overall fit (likelihood ratio χ^2^ = 122.13, *p* < 0.0001; pseudo R^2^ = 0.47).

Table 4 presents results for the final model. The results in the final analytic sample, confirmed to have met ATS standards for spirometry performance were compared with the full study sample results (likelihood ratio χ^2^ = 113.50, *p* < 0.0001) and were found to be consistent. Interactions of provider type with items and item-response weights were not statistically significant, indicating that items were not differentially predictive across provider type, and scoring weights functioned equally for general practice and specialist sites. The mean, median, and 5th and 95th percentiles for the 1000 bootstrap sample regression model results revealed little variation in model coefficients from the analytic sample results.

**Table 4:** Summary of logistic regression results

			Analytic Sample (N = 294)		
			
Item	Response	Item-Response Weights	Item OR (95% CI)	Response Options ORs	OR, CIs
During the past 4 weeks, how much of the time did you feel short of breath?	None of the time	0	1.07	—	—
	A little of the time	0	(0.75, 1.53)	1.27	0.38, 4.28
	Some of the time	1		0.88	0.26, 2.98
	Most of the time	2		1.13	0.30, 4.24
	All of the time	2		0.40	0.06, 2.63
Do you ever cough up any “stuff,” such as mucus or phlegm?	No, never	0	0.91	—	—
	Occas. colds	0	(0.72, 1.15)	1.49	0.49, 4.56
	Few days a month	1		1.84	0.54, 6.22
	Most days a week	1		1.48	0.43, 5.04
	Yes, every day	2		1.85	0.57, 6.05
Please select the answer that best describes you in the past 12 months. I do less than I used to because of my breathing problems.	Strongly disagree	0	1.64*	—	—
	Disagree	0	(1.25, 2.16)	0.77	0.23, 2.58
	Unsure	0		0.89	0.22. 3.54
	Agree	1		2.28	0.80, 6.54
	Strongly agree	2		6.78†	1.96, 23.47
Have you smoked at least 100 cigarettes in your ENTIRE LIFE?	No	0	5.80*	—	—
	Yes	2	(2.81, 11.96)	5.80*	2.68, 12.58
How old are you?	Aged 35 to 49 y	0	2.68*	—	—
	Aged 50 to 59 y	1	(1.92, 3.74)	2.85	0.78, 10.41
	Aged 60 to 69 y	2		6.80†	1.98, 23.35
	Aged 70+ y	2		23.80*	6.84, 82.83
Overall model fit:			LR test: 122.13, 16 df, *p* < 0.0001		

OR: odds ratio; CI: confidence interval; Occas.: Occasionally; LR: likelihood ratio; df: degrees of freedom.

* *p* < 0.001

† *p* < 0.01.

### Scoring and Screening Accuracy

**Figure 1:**
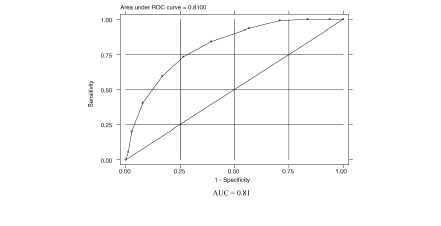
ROC curve: COPD-PS™ score and AO diagnosis. ROC: receiver operating characteristic; AUC: area under the curve; COPD-PS: COPD Population Screener; COPD: chronic obstructive pulmonary disease.

Of the 3 techniques used to evaluate scores, the weighted-sum technique produced the best results for discriminating between patients with and without AO. Each item response was assigned a value of 0, 1, or 2, depending on the relative contribution of the response to identifying AO, and response values were summed across the items to produce a scale score ranging from 0 (unlikely to have AO) to 10 (likely to have AO). Table 5 presents COPDPS mean scores by selected patient characteristics. Figure 1 presents the ROC curve associated with the final COPD-PS score. The AUC corresponding to a model with 5 individual screener items was 0.86, and for the total score it was 0.81. Based on sensitivity (59.6%) and specificity (83.2%) for the total score, the positive likelihood ratio (LR+) for the survey was 3.56.

**Table 5:** COPD-PS™ mean scores by selected patient characteristics

Patient Characteristics	Mean Score (SD)
Total population	5.04 (2.58)
Non-White	3.35 (2.61)
White	5.43 (2.41)
Male	5.36 (2.50)
Female	4.84 (2.61)
No AO	3.99 (2.35)
AO (all severity levels)	6.76 (1.94)
Mild AO (GOLD stage I)	6.33 (2.29)
Moderate/very severe AO (GOLD stage II+)	6.83 (1.89)
General practice site	3.99 (2.70)
Specialist site	5.63 (2.31)

COPD-PS: COPD Population Screener; SD: standard deviation; AO: Fixed airflow obstruction; COPD: chronic obstructive pulmonary disease; GOLD: Global Initiative for Chronic Obstructive Lung Disease.

Table 6 summarizes the performance of the COPD-PS score in identifying patients with AO across multiple score levels. In these analyses, screener scores were dichotomized to allow an analysis of cut-point performance relative to AO diagnosis. Lower cut-point scores were associated with higher sensitivity and lower specificity, while higher scores produced lower sensitivity and higher specificity. A cut-point in the range of 5 to 6 provided a good trade-off between sensitivity and specificity, as well as high correct classification rates for AO diagnosis.

**Table 6:** Performance of COPD-PS™ cut-point scores in screening for AO

Cut-Point Score	Odds Ratio	Sensitivity (%)	Specificity (%)	Positive Predictive Value (%)	Negative Predictive Value (%)	Percent (%) Correctly Classified	Area Under ROC Curve
≥ 4	11.10	93.6	43.3	50.3	91.7	62.4	0.68
5	8.34	84.4	60.7	56.8	86.4	69.7	0.73
6	7.69	73.4	73.6	63.0	81.9	73.5	0.74
7	7.29	59.6	83.2	68.4	77.1	74.2	0.71
8	7.92	40.4	92.1	75.9	71.6	72.5	0.66
9	8.75	20.2	97.2	81.5	66.5	67.9	0.59
Continuous Score	1.72	59.6	83.2	68.4	77.1	74.2	0.81

COPD-PS: COPD Population Screener; COPD: chronic obstructive pulmonary disease; AO: fixed airflow obstruction; ROC: receiver operating characteristic.

### Reliability

Table 7 presents mean scores for the analytic sample and by AO diagnosis for a random subsample of 57 patients completing the study surveys at entry and 2-week follow-up. For the analytic sample, the test-retest Pearson's product-moment correlation was 0.91 and the intraclass estimate was 0.91. Test-retest reliability estimates were slightly lower within each diagnostic group (AO and no AO), but the sample sizes may have been too small for accurate estimation.

**Table 7:** Test-retest reliability of COPD-PS™ scores

	Time 1 Mean (SD)	Time 2 Mean (SD)	Pearson's Test-Retest Reliability	Intraclass Coefficient Reliability
Total Sample (N = 57)	5.42 (2.5)	5.42 (2.4)	0.91	0.91
No AO (N = 36)	4.36 (2.2)	4.56 (2.4)	0.89	0.88
AO (N = 21)	7.24 (1.8)	6.90 (1.8)	0.88	0.86

COPD-PS: COPD Population Screener; SD: standard deviation; COPD: chronic obstructive pulmonary disease; AO: fixed airflow obstruction.

### Empirical Validation

Convergent and discriminant validity results are presented in Table 8, where scales are arranged in order from those designed to assess more physical aspects of health to those measuring mental health (higher SF-12v2 scores represent better health while higher COPD-PS scores represent higher likelihood of having AO). COPD-PS scores had higher correlations with scales designed to measure physical functioning, role limitations due to physical health, and general health status. Lower correlations were found between COPD-PS scores and scales designed to assess well-being domains, such as mental health.

**Table 8:** Association of COPD-PS™ score and SF-12v2 scores

	Correlation With COPD-PS
PCS-12	−0.47
Physical Functioning	−0.50
Role–Physical	−0.50
Bodily Pain	−0.12
General Health	−0.39
Vitality	−0.28
Social Functioning	−0.23
Role–Emotional	−0.23
Mental Health	−0.10
MCS-12	−0.06

COPD-PS: COPD Population Screener; SF-12v2 Health Survey: 12-item, short-form, general-health-status assessment; PCS-12: physical health summary measures of the SF-12 Health Survey; MCS-12: mental health summary measures of the SF-12 Health Survey.

Table 9 presents results of known-groups validity analyses. COPD-PS mean scores were significantly higher for patients with AO than without (6.8 vs 4.0, *p* < 0.0001). Scores for patients with mild AO were significantly higher than for patients with no AO (*p* 7lt; 0.01), but the difference between AO severity groups was not statistically significant. Patient- and physicianreported COPD also produced significantly higher mean scores. Bronchodilator use and overnight hospitalization were associated with significantly higher COPD-PS scores. Results from concurrent validity analyses demonstrated that the COPD-PS score predicted physician- reportedCOPDas well as spirometrybased diagnosis (model likelihood ratio χ^2^ = 148.57, *p* < 0.0001). AUC for the analytic sample was 0.88, for the continuous score sensitivity was 66% and specificity was 89%. Eighty percent of patients were correctly classified.

**Table 9:** Comparison of COPD-PS™ mean scores across patient variables

AO Comparison Variable	N	Mean	SD	*T*
COPD diagnosis				
No AO: FEV_1_/FVC > 70%	178	4.0	2.3	
AO: FEV_1_/FVC < 70%	109	6.8	1.9	−10.79*
AO severity				
No AO	178	4.0	2.3	
Mild AO: FEV_1_ > 80% predicted	15	6.3	2.3	−3.70†
Moderate/very severe AO: FEV_1_ < 80% predicted	94	6.8	1.9	−10.79*^1^ −0.92^2^
Practice type				
General practice	103	4.0	2.7	
Pulmonologist	185	5.6	2.3	−5.43*
Self-report				
No COPD	175	3.8	2.2	
Have COPD	111	7.0	1.7	−13.93*
Physician-report				
No COPD	175	3.7	2.1	
Have COPD	112	7.1	1.7	−14.88*
Use of bronchodilators				
No use in past 4 weeks	101	3.3	2.2	
Use in past 4 weeks	186	6.0	2.3	−9.71*
Work/school loss in past 4 weeks				
Did not miss due to breathing problems	116	3.9	2.4	
Missed 1+ days due to breathing problems	17	4.8	2.9	−1.36
Hospitalization in past 3 months				
No overnight hospitalization due to breathing problems	261	4.9	2.6	
1+ overnight hospitalizations due to breathing problems	19	6.5	2.7	−2.65†

COPD-PS: COPD Population Screener; COPD: chronic obstructive pulmonary disease; AO: fixed airflow obstruction; SD: standard deviation; FEV_1_: forced expiratory volume in 1 second; FVC: forced vital capacity.

* *p* < 0.001

† *p* < 0.01.

1 Moderate/very severe AO vs No AO.

2 Moderate/very severe AO vs Mild AO.

## DISCUSSION

Simple tools are needed to help identify persons who have COPD. A brief, easy-to-complete questionnaire based on patient-reported information can serve as a first-level screen. A screening assessment may help clinicians identify patients at risk for COPD, prompting clinical review and spirometric assessment, which are necessary to confirm a diagnosis. Furthermore, dissemination of a COPD screener in the general population might encourage individuals with pulmonary symptoms to visit their physician. This study represents the development and initial validation of a simple, reliable, self-scored COPD screening questionnaire. Three COPD-related items (breathlessness, productive cough, and activity limitation), a smoking history item (100 or more cigarettes smoked in lifetime), and an age item were used to construct the COPD-PS questionnaire. A weighted-sum score of these items resulted in a questionnaire that discriminated between patients with and without AO, and could be easily adapted to an electronic format for simple scoring.

The hallmark symptoms of COPD are dyspnea, chronic cough, chronic sputum production, and a history of exposure to risk factors, such as smoking (3). The COPD-PS identifies these symptoms and risks, as well as considers age as a screening factor for COPD. The COPD-PS is unique in that it is a self-administered, self-scored questionnaire that utilizes a scoring system to predict spirometry-based diagnosis of the disease. Individual COPD-PS items were not all significant predictors of COPD in a multivariate logistic model, but their combination produced a well-fitting model and a final score that performed well in tests of sensitivity and specificity. Scores met generally accepted standards for individual-level reliability (24), suggesting that they are not only appropriate for group-level inferences but also can be applied reliably at the individual level.

The clinician working group tested several different versions of items that assessed concepts typically associated with COPD. This allowed identification of questions that could be easily understood and completed by patients across a range of literacy skills. Some particular item wordings had a stronger relationship to COPD than others. Surprisingly, several concepts identified by clinicians as related to COPD—cough, frequent colds or bronchitis, and chest congestion or wheezing—did not serve to distinguish between patients with AO and those without AO. Although these are likely important aspects of the disease, self-reporting of cough, colds, and chest congestion did not improve the differentiation between AO and no AO when breathlessness, phlegm, functional limitations, smoking status, and age were considered. This may relate to the similarity shared by several pulmonary conditions for these nondistinguishing symptoms.

The group studied the contribution of smoking status, measured in multiple ways, to prediction of COPD. Each smoking variable performed similarly in discriminating between patients with and without AO when tested in combination with other screening items. In our attempt to develop an easy-to-complete questionnaire that did not require complicated calculations, the combination of explanatory power and ease of administration resulted in the selection of a single-item smoking measure for the COPD-PS. Other state (25, 26) and national (27, 28) surveys have used a similar “smoked at least 100 cigarettes” item. Its inclusion in the COPD-PS allows for comparison of smoking rates with nationally published figures.

We demonstrated validity of the COPD-PS through multiple concurrent and construct approaches. Higher COPD-PS scores can generally be interpreted as indicating an increased likelihood of AO. Lower cut-point levels were associated with greater detection of patients with AO (higher sensitivity) but also included some patients not having AO (low specificity). The opposite was true at higher scores. Thus, selection of a particular cut-point value can be adapted for a particular application. For example, if the goal is to identify as many individuals with potential COPD as possible, with less concern for including those who may not have the disease, a score corresponding to high sensitivity and lower specificity can be considered. This could result in significant health care resource expenditure. On the other hand, if an application requires a higher level of certainty that all identified individuals do have COPD, a score associated with higher specificity is desirable. As such, the optimal scoring utilized should be adapted to the situation and with regards to the health care utilization implication of this decision.

In contrast to this self-report questionnaire, some earlier COPD screening tools require clinical information from the medical record (29, 30) or previous report of a physician diagnosis of COPD (31) for scoring. Others require interviewer administration, include complicated calculations or skip patterns that make it difficult to self-administer, or are intended only for a particular population, such as current or former smokers (32). The value of self-report surveys relative to spirometry has been demonstrated for COPD case finding (32–34). van Schayck et al. identified an optimal combination of items from NHANES-III for distinguishing between those with and without COPD, but because that study did not include reversibility testing, it is unclear how well results will generalize to COPD diagnoses based on GOLD definitions of the disease (35).

Price et al. generated an 8-item COPD questionnaire developed in patients with a positive smoking history and no previous respiratory diagnosis identified through 2 primary care physician office sites (United Kingdom and United States) (32). This questionnaire included items related to age group, body mass index, pack-year history, and symptoms and featured a scoring system suitable for use in primary care settings. Scores on this case-finding tool were separated into 3 “zones,” indicating increased, unchanged, and decreased likelihood of COPD compared with the total study population (which as a whole has an increased risk of COPD due to inclusion criteria); patients with a higher likelihood should be referred for spirometry (36). Although some of the items are similar to those of the COPD-PS, its derivation suggests that this instrument is better suited for use in a physician's office in higher risk populations.

Similarly, Freeman et al. identified 4 features (age, cough, dyspnea, and wheezing) that identified patients with COPD among a primary care population with a positive smoking history, history of use of respiratory medications, or a history of asthma (33). Calverley et al. developed a population-based screening questionnaire for COPD retrospectively, using NHANES III data (12). The COPD-PS differs from these other instruments because it can be used in a broader group of individuals, regardless of smoking history or known presence of respiratory problems. Although other instruments include age and symptom-based items, the COPD-PS also contains a disease-impact item, which allows the patient to describe activity limitations due to breathing problems.

A limitation of this study includes the definition for COPD. Although the screener identified patients with AO and not necessarily COPD, it does identify patients for whom spirometry would be indicated and in whom additional clinical evaluation would be required to confirm a COPD diagnosis. Furthermore, the important contribution of the smoking item, the limited bronchoreversibility of patients with AO, and the high correlation with physician-reported COPD strongly support that cases with AO identified had a COPD diagnosis. In fact there was only fair concordance between subject reported or clinician diagnosed COPD and confirmed COPD; one in five spirometry-identified COPD patients did not report having a previous COPD history by either self-report or clinician diagnosis.

An additional limitation reflects the source of the analytic sample studied. Patients were recruited from a clinical setting to allow postbronchodilator spirometric assessment. In addition to patients from specialist sites, patients from general practice sites were enrolled, with the intention of accessing individuals who were not already seeing a pulmonary specialist for respiratory symptoms. Similarly, recruited patients were being seen for routine, previously scheduled, office visits to maximize generalizability. It is important to realize that the prevalence of COPD was higher in the cohort studied than would be anticipated in general population samples, which may have increased the positive predictive value of the COPD-PS. Validation in an independent general population sample is required to define the operating characteristics in this setting.

Patients were visiting their physician for other medical problems, which might confound the diagnosis of COPD. An additional limitation was the low rate of adherence to ATS standards for spirometry testing. More than half of the tests carried out in general practice sites did not meet either strict reproducibility or acceptability standards for 3 tracings, according to external review. However, even in 2 of the 4 specialist sites, there were many tracings that were excluded.

Despite this, results of analysis in the analytic sample were similar to the results for the entire study population. Importantly, the lack of adequate spirometry performance in a majority of subjects supports the need for aggressive training in spirometric testing if a screening instrument such as the COPDPS is to be widely employed. Previous investigators have confirmed that a majority of maneuvers performed in the primary care setting without access to training failed to meet reproducibility criteria (37). This group and others have confirmed improvement in spirometry quality with limited training (38). Using primary care physicians to identify early COPD with spirometry will require ensuring that spirometry is properly performed (6).

Validation studies are needed to confirm the performance of the COPD-PS questionnaire among individuals in community settings. Future studies may choose to evaluate whether additional item content can improve the performance of a brief COPD screener. Cognitive interviews or focus groups with patients or individuals from the general population may provide additional information about the acceptability and readability of the COPD-PSquestions. Although a secondhand smoke item approached statistical significance in our analytic sample, future studies evaluating this item in nonsmokers may be of interest. The developmental survey did not include self-report items for calculating body mass index. Items that separate asthma and COPD could also be evaluated. The COPD-PS can be evaluated for its utility as a first-stage screener in various settings, such as population screening, clinical practice, disease management, or population-based research.

## Notes

Funding was provided by Boehringer Ingelheim Pharmaceuticals, Inc. and Pfizer Inc.

Members of the COPD-PS Clinician Working Group include Roderick Bartlett, MD, Hannibal Clinic, Hannibal, MO; P. James Connor, MD, Creighton University Medical Center, Omaha, NE; Claudia Cote, MD, VA Medical Center, Bay Pines, FL; Clare Hawkins, MD, MSc, San Jacinto Methodist Hospital, Baytown, TX; Mary Issac, MD, MPH, St. Joseph's Community Care Hospital, Tampa, FL; Fernando J. Martinez, MD, MS, University of Michigan, Ann Arbor, MI; Phillip Menashe, MD, Yakima Chest Clinic, Yakima, WA; Anna W. Parkman, RRT, PhD, Ohio Dominican University, Columbus, OH; Michael A. Russoniello, MD, St. Clares Hospital, Denville, NJ; Frederic D. Seifer, MD, Takoma Adventist Hospital, Greeneville, TN.

## Appendix

**Appendix A:** Item content of variables tested for inclusion in the COPD-PS™ survey

Conceptual Domain	Item Content
Dyspnea	During the past 4 weeks, how much of the time did you feel short of breath?
	Do you get out of breath with activity?
	On average, breathing problems usually keep me awake at night… (frequency)
	Over the last year, I have had shortness of breath:
	During the past 4 weeks, how often did breathing problems limit your ability to exercise as much as you would like?
	In the past 4 weeks, how often have you felt short of breath under the following conditions? (When lying down flat; When sitting or resting; Getting dressed; When walking less than one block; Bending over; When climbing one flight of stairs; With heavy exercise or manual work (running, cycling, swimming fast))
	Do you get out of breath more easily than others your age?
Cough	Over the last year, I have coughed:… (frequency)
	In the past 4 weeks, how often have you had any of the following? (Coughing first thing in the morning; A cough that just won't go away; A cough that makes your chest hurt; Coughing “attacks” when you exercise; A cough that wakes you up at night; A need to cough to clear your chest)
	How often in the past 4 weeks have you had a nagging cough?
Phlegm	Do you ever cough up any ‘stuff’, such as mucus or phlegm?
	How often in the past 12 months have you brought up phlegm or mucus first thing in the morning?
	Please select the answer that best describes you in the past 12 months. I have to clear my chest of stuff when I wake up in the morning.
Colds/bronchitis	Please select the answer that best describes you in the past 12 months. (I get a lot of chest colds; When I get a cold it really stays with me; My colds last for weeks rather than days; I seem to catch a cold more easily than other people do; I get bronchitis at least once every winter; I have frequent bouts with bronchitis)
Chest Congestion/Wheezing	In the past 4 weeks, how often have you had any of the following? (A feeling like something might be “stuck” in your chest or lungs; A feeling of heaviness in chest; Noisy breathing when you sleep; Chest congestion; Noisy breathing during the day (gurgling, bubbling, rattling)
Functional Impact	I do less than I used to because of my breathing problems.
	In the past 4 weeks, how much did breathing problems limit your usual activities or enjoyment of everyday life?
	In the past 4 weeks, how much of the time did you have difficulty in performing work or other daily activities because of breathing problems?
	In the past 4 weeks, how often: (did breathing problems limit you in performing your usual work activities, including housework, work, school or social activities; did breathing problems keep you from socializing; did you feel fed up or frustrated because of breathing problems; did breathing problems leave you too tired to do work or daily activities; did breathing problems keep you from getting as much done at work or at home; did breathing problems make it difficult for you to focus your attention on other things?)
Personal Characteristics	Do you find that certain strong smells such as exhaust fumes, cigarette smoke or paint fumes affect you: (extent of effect)
	Smoking history (Current and past status, pack-years)
	Do you have a family history of emphysema or chronic lung problems? Have you been exposed to tobacco or other kinds of second-hand smoke at home or work for extended periods of time?
	Do you live with someone who smokes?
	Have you been exposed to dust, gases, or dirty air at work?
